# Evidence for a Cultural Mindset: Combining Process Data, Theory, and Simulation

**DOI:** 10.3389/fpsyg.2021.596246

**Published:** 2021-09-10

**Authors:** LaTasha R. Holden, Michelle LaMar, Malcolm Bauer

**Affiliations:** ^1^Department of Psychology, Florida State University, Tallahassee, FL, United States; ^2^Florida Center for Reading Research, Tallahassee, FL, United States; ^3^Educational Testing Service, Princeton, NJ, United States

**Keywords:** cross-cultural competence, mindset, decision making, computational model, Markov decision process, simulation study

## Abstract

Despite large literature on Cross-Cultural Competence (3C) there is a gap in understanding learning processes and mechanisms by which people arrive at successful 3C. We present a novel perspective for 3C learning and decision-making in innovative assessment contexts. We use Mindset theory (i.e., believing ability is fixed or changeable) because it is shown to be a powerful motivator for general learning and performance and in cross-cultural contexts. We propose the notion of cultural mindsets – beliefs, affect, and cognition that govern how people adapt, learn, and update cultural information. To understand how cultural mindset affects learning and performance, we apply computational cognitive modeling using Markov decision process (MDP). Using logfile data from an interactive 3C task, we operationalize behavioral differences in actions and decision making based on Mindset theory, developing cognitive models of fixed and malleable cultural mindsets based on mechanisms of initial beliefs, goals, and belief updating. To explore the validity of our theory, we develop computational MDP models, generate simulated data, and examine whether performance patterns fit our expectations. We expected the malleable cultural mindset would be better at learning the cultural norms in the assessment, more persistent in cultural interactions, quit less before accomplishing the task goal, and would be more likely to modify behavior after negative feedback. We find evidence of distinct patterns of cultural learning, decision-making, and performance with more malleable cultural mindsets showing significantly greater cultural learning, persistence, and responsiveness to feedback, and more openness to exploring current cultural norms and behavior. Moreover, our model was supported in that we were able to accurately classify 83% of the simulated records from the generating model. We argue that cultural mindsets are important mechanisms involved in effectively navigating cross-cultural situations and should be considered in a variety of areas of future research including education, business, health, and military institutions.

## Introduction

Working and interacting effectively with individuals from different cultural backgrounds has become increasingly important in many workforce and educational settings. With projections for religious and ethnic/racial diversity and international immigration in the United States continuing to rise (Passel and Cohn, 2008; Bump, 2015), the implications of a multicultural society and world are more important than ever. Through utilizing a cross-cultural perspective, we better understand how quickly and efficiently people learn to function within and across cultures and situations.

Successful performance in cross-cultural environments is highly relevant and valuable in a variety of fields, including: education, healthcare, and business. In education, understanding different cultural backgrounds can make teachers more informed, relatable, and effective. Research suggests that cross-cultural competency is an important skill for effective teaching, as culturally responsive teaching can increase student educational outcomes and decrease inappropriate placement and referrals (Gay, 2000; Goe et al., 2008; Lim, 2014). In healthcare, implicit biases and cultural misunderstandings can interfere with decision-making among healthcare professionals – contributing to the disparity in negative health outcomes suffered by ethnic minorities (Mayberry et al., 2000; Institute of Medicine Committee on the Health Professions Education Summit, 2003; Health Policy Institute, 2004; National Center for Chronic Disease Prevention and Health Promotion, 2016). In business, increasingly, our global economy depends on the functioning of highly diverse teams. In some cases, culturally homogenous groups have been shown to be more productive than culturally heterogenous ones (DiStefano and Maznevski, 2000). These productivity gaps arise from barriers to shared understanding and failures to competently tap into the range of valuable skills that diverse groups offer (DiStefano, 2003). However, more diverse teams are shown to outperform less diverse ones only after acknowledging and working around differences and establishing team norms (DiStefano, 2003). Taken together, these findings highlight the importance of better understanding the processes and mechanisms of cross-cultural interactions that enable successful performance in a variety of fields.

### Cross-Cultural Competence

Though definitions vary, cross-cultural competence, or 3C, is thought of as a “set of knowledge, skills, and affect/motivation that enables individuals to adapt effectively in cross-cultural environments” (Abbe et al., 2007, p. vii). A great deal of research has been dedicated to improving the understanding of what contributes to effective cross-cultural performance and identifying the best means by which to measure it (Chiu et al., 2013; Griffith et al., 2016; Liu et al., 2018). To date, a multitude of definitions, theories, frameworks, and assessments have been developed on the topic of cross-cultural performance (see Gallus et al., 2014), however, most work has focused on the operationalization of the construct (or, in some cases, constructs) believed to influence and predict effective cross-cultural performance. Previous research notes the importance of resolving issues related to definitions and measurement, urging further exploration of the “antecedents, core characteristics, and consequences (Chiu et al., 2013) of 3C.” Indeed, much of the 3C and performance research lacks clarification about how cross-cultural knowledge is accumulated and learned as well as how people differ in their abilities to adapt in cross-cultural interactions.

In terms of how people adapt to new environments, the knowledge component of 3C reflects an understanding of the broad, universal dimensions or patterns upon which most cultures are based (e.g., Murdock, 1945; Hofstede, 2001) – as opposed to discrete “facts” or behavioral scripts that likely apply only to a select number of groups or specific situations. Likewise, the skills component reflects both cognitive and metacognitive processes, such as perspective taking, emotion regulation, and hypothetico-deductive reasoning, all of which facilitate performance and adaptation, especially in environments that are unfamiliar or where cultural information is limited or unavailable. In order to better understand differences in the processes that drive successful and unsuccessful thinking and behavior in cross-cultural contexts, having a means for capturing the skills that facilitate performance, as well as the ways in which people behave when information is limited or ambiguous is especially important.

More recent work emphasizes the process-oriented or dynamic nature of 3C (see Thomas et al., 2008; Burrus et al., unpublished manuscript). In this sense, 3C is less something one *has* and more something one *uses* to learn about other cultures. Moreover, it is important to note that just because one is cross-culturally competent does not imply that they will adapt to or perform in a new culture with no errors. Indeed, cross-culturally competent individuals are just as likely to make mistakes as individuals who are less cross-culturally competent, especially early on in the adaptation process. The difference between these two types of individuals, however, lies in the degree to which each attends to, evaluates, and learns from those mistakes as a way to enhance their understanding and performance within that culture (see McCloskey et al., 2010). These are important considerations for our understanding of the complexity of cross-cultural competence and how it relates to differences in performance.

Although 3C has been researched widely, there is currently a gap in the literature in understanding the processes and mechanisms by which people arrive at successful 3C. There is limited research on the assessment of 3C through performance tasks. Chiu et al. (2013) report that only one major assessment of 3C involved behavioral measures as opposed to self-report or trait assessment methods. Although self-report and questionnaire techniques save time in administration they may not prove adequate in predicting future adaptation and adjustment (Klafehn et al., 2013) and are unable to provide detailed information about why there are breakdowns in successful cross-cultural communication.

To our knowledge, no 3C studies have aimed to more carefully consider the processes and mechanisms that clarify how differing person by situation contexts drive different patterns of behavior. We argue that the social psychological construct of *Mindset* (Dweck, 2006) is a useful theoretical framework for better understanding important mechanisms in cross-cultural interactions and performance. Specifically, we will use Mindset theory to help frame how the malleability of our cultural beliefs can impact how we learn, adapt, and perform. In order to better understand the cognitive process by which people behave in cross-cultural situations, we considered that people may have different initial beliefs and dispositions that influence affect, cognition, and behavior in these situations. Thus, we used Mindset theory to help develop and articulate cognitive differences that people bring to cross-cultural situations.

### Mindset Theory and Research

Basic research in social psychology helps frame how our beliefs about ourselves and others impact how we behave and perform (see Dweck, 2006). The implicit theories of ability framework, also called *Mindset* (Dweck, 1999) proposes that people’s beliefs about how changeable their abilities are can be classified on a continuum of fixed to malleable (Dweck and Leggett, 1988; Dweck, 2006). In seminal work on motivation and performance, Licht and Dweck (1984) proposed that students approach tasks differently based on their beliefs about the nature of their ability in different domains. They found that when presented with a learning task containing confusing information, “attributional style” had the largest impact on their performance; those with “mastery oriented” styles outperformed those with “helpless” styles. These results correspond with current work on implicit beliefs of intelligence – performance wise, those with “mastery” orientation styles align with incremental/malleabile theories of intelligence, whereas, “helpless” orientation styles align with entity/fixed theories of intelligence. Licht and Dweck’s results indicate that achievement differences emerge from differences in achievement orientations (i.e., fixed vs. malleable mindsets) in conjunction with the demands of specific skill areas (e.g., domain-specific abilities, interests, or aptitude).

The crux of mindset theory involves beliefs about control: those with fixed mindsets tend to believe that people have less control over their abilities, whereas those with malleable mindsets believe that people have the power to enhance their abilities, if desired (Dweck, 2011). In terms of performance, Dweck (2011) asserts that for people with fixed mindsets their motivation is around validating and supporting fixed beliefs about ability through their performance, whereas for malleable mindset people, the focus is on ability enhancement through seeking out learning opportunities.

In an application of mindset theory to culture, Dweck (2012) investigated the attitudes of Israelis and Palestinians with hopes for using mindset interventions to bring about peace between the groups. Results revealed that Israelis’ fixed mindsets were correlated with holding negative attitudes toward Palestinians. Moreover, both Israelis and Palestinians were more likely to have positive attitudes toward the other group when they completed a mindset manipulation which involved reading about how groups in general are capable of change. This research suggests that having fixed mindsets may be associated with biases and negative reactions toward out-groups. Also, these findings provide evidence that manipulating one’s mindset could decrease bias and discrimination in cross-cultural contexts.

When looking at the role of mindset across Eastern and Western cultures, those who have more fixed mindsets tend to attribute the underlying cause of others’ behavior to dispositional rather than situational factors (see Chiu et al., 1997). In general, those who hold more fixed views tend to be more likely to subscribe to attitudes congruent with stereotypes about others. People with more fixed mindsets tend to view out-groups as more homogenous than their in-group, and in turn, show more biased behavior toward those with different group identities than their own (Levy et al., 2001).

In additional work focused on the role of mindset and intercultural contact, Chao et al. (2017) examine predictors of ones’ ability to adapt and function in different cultural settings (deemed cultural intelligence) over time by recruiting students in an international exchange program. They found that for students with more fixed cultural beliefs this led to greater sensitivity to rejection in terms of perceived likelihood of being rejected and, also related to feelings of anxiety in intercultural situations. Additionally, for those with more fixed cultural beliefs, increased rejection sensitivity predicted worse cross-cultural adjustment and comfort in cross cultural contexts, which in turn predicted lower cultural intelligence.

Expanding upon empirical work with mindset and intercultural contexts, Lou and Noels (2019) implement both correlational and experimental approaches to understanding how mindsets about language learning impact rejection sensitivity and cultural adaptation for language learners in non-native cultural settings. They found that more fixed mindsets about language caused stronger language-based rejection sensitivity, higher levels of anxiety and lower levels of cultural adjustment. Even when participants’ levels of perceived language competency were controlled for, the effect of fixed mindset on rejection sensitivity remained. However, they also found that the more malleable mindsets were more motivated to improve and more likely to seek out social encounters with native speakers – suggesting that more malleable mindsets may help people reconsider negative perceptions from native speakers, making them more likely to engage with native speakers in spite of potential worries about their actual or perceived competency. This underscores the potential benefits of malleable mindset in that people are more willing to engage and learn, and more open to engaging in cross-cultural contexts where they may not feel that they have mastery of a skill or ability. However, both Lou and Noels (2019) and Chao et al. (2017) heavily relied on self-reported behaviors and did not examine actual performance.

Taken together, this review demonstrates that Mindset theory provides appropriate and useful framing for thinking about different patterns of behavior in cross-cultural situations. Specifically, Mindset theory aids our understanding of how implicit beliefs drive behavior and provides a useful lens for thinking about what people pay attention to and how they evaluate and learn information in cross-cultural settings. Our work aims to incorporate different models of cognition and decision making based on having more fixed or malleable mindset and we in turn, examine participants behavior and performance through a cross-cultural assessment game. Our work builds on previous studies by further examining the mechanisms involved in arriving at successful cross-cultural interactions, allowing us to better understand how fixed and malleable mindsets are likely to differ in their patterns of thinking and behavior.

Next, we will review literature on the role of mindset and cultural competence in helping individuals learn, adapt, and perform in non-native settings.

### Cultural Mindsets

Depending on the cultural norms and rules within a group (and on awareness of these rules) people may engage in patterns of behavior based on their understanding of the culture along with their own predisposed beliefs. We propose a new theoretical model called *cultural mindset* defined here as the set of beliefs and attitudes that people bring to cross-cultural contexts that govern the ease with which people adapt, learn, and update cultural information^1^. Cultural mindset involves the willingness to learn and adjust cultural attitudes (including affect, cognition, and behavior) in order to adapt to the cultural norms of a situation; it is not simply the process of changing cultural beliefs. We expect that cultural mindset can reveal the mechanisms that explain how differences in beliefs drive differences in observed behavior within a cross-cultural context.

While we expect cultural mindset to be a continuum, we will focus our discussion and exploration of the concept by defining two distinct cognitive profiles that can be thought of as endpoints on the spectrum: a fixed and a malleable cultural mindset. The hallmark of a fixed cultural mindset is the belief that people’s cultural understanding and attitudes are unchanging and unchangeable. This belief extends to thinking about cultural norms and culturally appropriate behavior, which the fixed cultural mindset individual would assume to be set and unambiguous. It is likely that this individual would project his/her own culture onto members of other cultures or expect them to behave in a similar fashion based on cultural stereotypes. In contrast, a malleable cultural mindset would embrace the idea that cultural norms can be learned and that culture itself is a complex, nuanced, construct. Individuals with the malleable cultural mindset are likely to consider the possibility of cultural differences and be more willing to adapt their thinking and behavior. Because the goal of the current work is to better understand 3C and performance, having better ways for identifying and understanding successful cross-cultural encounters from a performance-based perspective is essential.

### Statement of Purpose

The purpose of the current paper is to move the field forward by arguing for the need and value of a cultural mindset construct and demonstrating a novel methodology for exploring the implications of cultural mindset on performance in cross-cultural contexts. Dweck (2006) proposes that whether people have fixed or changeable beliefs about ability predicts different patterns of action and behavior, and in turn, how they perform on challenging tasks. In this paper, we propose that Mindset is a key aspect that drives behavior in cross-cultural interactions in what we call different kinds of *cultural mindsets*. For example, we argue that cultural mindsets can influence and drive different patterns of behavior in social situations which correlate with either successfully or unsuccessfully achieving a special task goal.

Additionally, because 3C research has identified the need for a closer examination of mechanisms that influence performance, we demonstrate that the theory behind cultural mindset mechanisms can be expressed and tested computationally. We developed cognitive profiles for two cultural mindsets based on data from student performance in cognitive lab studies. We then use these profiles to build computational cognitive models of decision-making, which allow us to simulate performance within a cross-cultural game as if a computer agent was playing the game. Cross-cultural competence is situational, and this presents a unique challenge for studying it. However, digital experiences such as a cross-cultural learning game allows us to assess and study 3C in a richer and more authentic way. Through the cross-cultural game we use online experience to study 3C in a controlled environment with simulated agents – later using computational cognitive models to better understand the mechanisms involved in successful 3C. As the computational models embody the theorized mechanisms which drive cultural mindset, the actions generated by the different models can be used to test these theories. We use simulation studies to make and later test specific behavioral predictions regarding the cultural mindset construct, arguing that this is a novel and fruitful approach to investigating the roles of cognition and performance in cross-cultural scenarios. A goal of the present work is to explore the effects of different cultural mindsets through modeling the cognitive implications of the theory. Next, we describe the 3C assessment task and how cultural mindsets might affect performance on the task. We then detail the objectives of our study, followed by how the specific cultural mindsets were modeled computationally.

## Assessment Instrument

We explore behaviors within a cross-cultural setting using an interactive narrative game which was designed to assess 3C (Gjicali et al., 2020, also see Samuel et al., 2018). The player plays the part of a foreigner participating in a contest. Within the game, the player is given objective tasks; however, task completion requires interaction with and cooperation from a variety of non-player characters (NPCs). Interactions are dialog-based, with each player’s turn involving the selection of an utterance from 2 to 6 possible responses dynamically created from the state of the simulation. The NPC then reacts to the player’s dialog through facial expressions and verbal responses. To make this an assessment of cultural learning rather than cultural knowledge, a fictitious culture was designed for the game.

### Simulated Culture

The social simulation game included two important core cultural dimensions (a) hierarchy-egalitarianism and (b) individualism/collectivism (Nolan et al., 2014). Here, we focus on one facet of the culture, specifically, we defined a limited artificial culture in which hierarchy matters. This manifested as differences in status in which (1) older people (65+) and younger people (0–17) are of high status, while people in the middle (roughly age 18–64) are of lower status. Cultural manifestations of status include how people prefer to be greeted and asked for information (high status people prefer to be addressed formally while lower status prefer informally).

### Task Design

**FIGURE 1:**
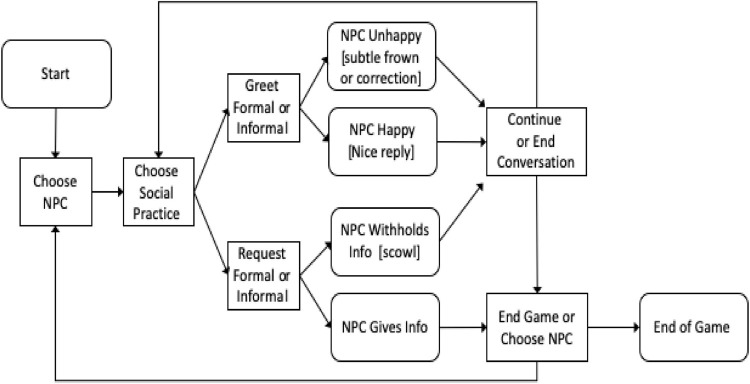
Flow diagram for the simple version of the Ask-for-Water task. Square boxes represent choices that the player makes and round boxes indicate game states.

The assessment task involves a scenario in which the player is new to a village and is trying to locate a water source (we refer the reader to Gjicali et al., 2020 for more detailed information about the assessment task). To achieve this goal, the player must initiate interactions – greeting strangers in a culturally appropriate way, also being responsive to cultural feedback should they make an error. The task and underlying simulation are designed such that players select from a set of possible responses each time they wish to act or respond (e.g., by selecting a formal or informal greeting; a polite request vs. a demand). Some responses are more or less appropriate given both the artificial culture and the context in which the interaction is taking place. As such, conversations require players to pick up on context and meaning when speaking with the NPCs (for a review on pragmatic competence see Laughlin et al., 2015) as well as responding in an appropriate manner. In the scenario, a player who initiates an interaction inappropriately, but adjusts his or her behavior in response to NPC feedback is demonstrating a higher level of cross-cultural skill than a player who continues to interact inappropriately despite receiving negative feedback. Figure 1 provides a diagram for the game.

**FIGURE 2:**
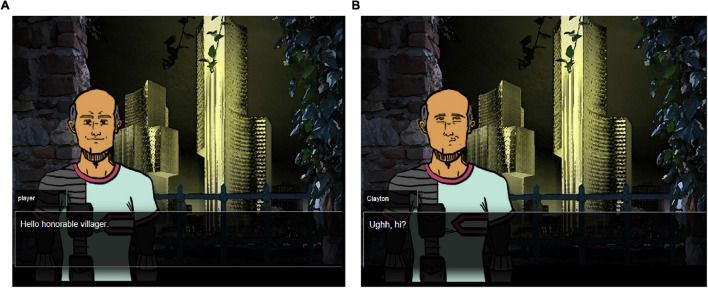
**(A)** Shows the positive response to the “Formal Greet” action as displayed in the game. **(B)** Shows the negative response to the “Informal Greet” as a subtle frown.

Play begins with six non-player characters (NPCs) on a map. The NPCs and their characteristics are described in Table 1. Selecting an NPC brings up their image and the available social practices players can choose to initiate interaction. In this task the players choose to greet or make a request. Once the player chooses a social practice they are given a set of dialog choices to implement that practice. For example, to greet NPCs they choose from “Hi” or “Hello honorable villager.” The NPC response is determined by a dynamic model of the NPC which includes personality characteristics and their current inclination toward the player (McCoy et al., 2014). Figure 2A (below) is the result of the player choosing “Hello honorable villager” to which the older NPC reacts positively.

**TABLE 1:** The six NPCs in the game and their characteristics.

NPC Name	Gender	Age	Profession
Stan	male	young	Student
Belle	female	young	Mayor
Emma	female	middle-aged	Sheriff
Mick	male	middle-aged	Pilot
Brim	female	elder	Nanosmith
Clayton	male	elder	Farmer

At this point, the social simulator runs, calculating the desirability of the actions available to the NPC (i.e., Clayton, the Farmer). As expressed in Figure 1, there are two reactions from the NPC (negative greet if the greeting is inappropriately matched to the NPC’s status and positive greet otherwise). Across NPCs, three responses: “subtle frown,” “correction,” or “nice reply” are selected for the NPC by the social simulator, each associated with a different intent. Clayton, as an older person prefers to be greeted formally, and so will respond positively to a formal greeting (Figure 2A) and more negatively to an informal greeting (Figure 2B). Play continues, roughly following the interactions and player choices outlined in Figure 1 until an NPC provides directions to the water or does not. Players then choose a different NPC with whom to interact perhaps changing their beliefs about the underlying cultural knowledge based upon feedback (or not).

### Cultural Mindset Within the Task

We expect the difference between the fixed and malleable cultural mindsets lies in the degree to which each attends to, evaluates, and learns from mistakes as a way to enhance their understanding of and performance within a culture. We hypothesize that, because 3C involves learning in a new environment, and potentially revising prior beliefs, people with more of a fixed cultural mindset will perform less well on tasks that involve 3C than those with more of a malleable cultural mindset. In the context of the task we redefine the two mindset profiles below.

### Fixed Cultural Mindset

We expected that people with more fixed cultural mindsets use what we deem as a confirmatory reasoning strategy. First, we expected that those with fixed cultural mindsets project his/her own culture onto others. Then, they use confirmatory reasoning to search for evidence of culture that aligns with their own expectations. In turn, their main objective is to be efficient with achieving a task goal and this may come at the expense of learning. Thus, we expected to see relatively short interactions, conversations, and shallow engagement with other people in a new culture. We also expected that when it takes longer to complete a task goal than expected they will be more likely to quit and seek out new methods to more quickly achieve the task goal. Based on a confirmatory reasoning strategy, those with fixed cultural mindsets will have difficulty updating and incorporating new information into their beliefs. We expect this is because they will have difficulty seeing beyond their own experience and perspective (lack of effective perspective taking). Furthermore, the effortful process of updating and integrating new information into understanding and application in the current cultural context is expected to be relatively slow (as compared to a malleable cultural mindset person).

### Malleable Cultural Mindset

We expect that malleable cultural mindset people use a different strategy which we labeled the explore and learn strategy. This approach is outlined in terms of a different perspective compared to those with a fixed cultural mindset. For the malleable cultural mindset people, the goal is to explore and observe behavior. Based on receiving feedback they will incorporate it and update their beliefs easier than the fixed cultural mindset person. Also, we expect they will be relatively open in their beliefs and be open to updating. Based on the explore and learn strategy we expect malleable cultural mindset people to be flexible and open to task goals taking longer because gathering information is more rewarding than being more efficient (as compared to the fixed person). For example, we expect to see that they are willing to try different methods and paths of action that can be explored in order to learn about a new culture. And we also expect malleable mindset people to reflect on their own actions and regulate their behavior based on feedback. Having a more malleable cultural mindset will be associated with greater willingness to explore and update cultural beliefs more easily and often relative to the fixed cultural mindset person.

### Study Objectives

Given these mindset profile definitions, we use simulation studies to validate our formulation of the cultural mindsets and the mechanism by which we propose that they manifest in cross-cultural situations. As validity checks we propose several criteria that we expect to be true for our simulated players. *Criteria 1:* Malleable mindset players will be more successful at learning cultural norms of the game. We investigate this by comparing the initial cultural beliefs and the final cultural beliefs of the fixed and malleable players. *Criteria 2*: Fixed mindset players will have fewer conversations and fewer turns per conversation than malleable players. We investigate this by comparing the average number of actions overall and the average conversations started across fixed and malleable players. *Criteria 3*: Fixed mindset players will be more likely to give up in the process of achieving the task goal of finding water and more likely to switch conversational partners when unsuccessful. We investigate this by comparing the rates of game quitting and conversational quitting among the fixed and malleable players. *Criteria 4*: Fixed mindset players will be less likely to change their behavior after receiving negative feedback. We investigate this by comparing the rates of responsiveness to feedback from the NPCs and the rates of responsiveness to at least one incorrect use of formality type among fixed and malleable players. *Model Classification*: In addition to our validity criteria, we test model recoverability by classifying each simulated record as either fixed or malleable using maximum likelihood estimation and comparing the results to the actual generating model. *Exploratory Analysis*: Because the age of the NPCs was visually apparent during real-world gameplay and players may have pre-existing normative beliefs about age, we also explored whether the models would simulate differences in preferences for interacting with NPCs based on age. We hypothesized that older NPCs might be selected more based on potential pre-existing normative beliefs about age and ease of achieving the task goal.

## Methods

### Markov Decision Process Computational Models

**FIGURE 3:**
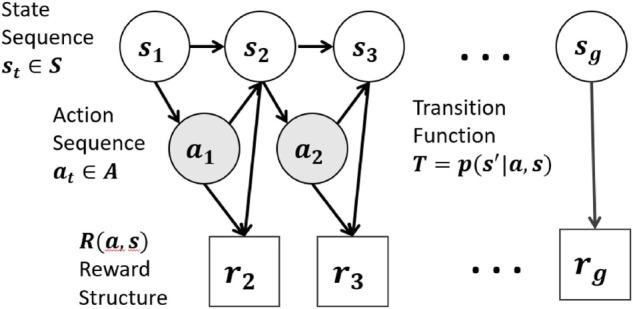
Markov decision process models a path through a problem space as a sequence of actions taken, where each action results in a cost or benefit (the rewards) and changes the state of the problem in some way, reflected in the state space. In many situations there is a goal state *s*_*g*_ which results in a large, positive reward.

In order to explore how mechanisms implied by the cultural mindset theory result in behavioral differences we developed generative computational cognitive models for the two contrasting mindsets: fixed and malleable. These models are coded using partially observable Markov decision processes (POMDP), an approach that models goal-directed decision making within a non-deterministic environment (Puterman, 1994). The Markov decision process software and all code, models and data are available upon request. The Markov decision process (MDP) is a statistical algorithm for choosing optimal actions based on a longitudinal cost-benefit analysis. While the optimization criterion can vary, most commonly the MDP is solved to maximize expected total rewards over a multi-step problem space. What counts as a reward is a configurable element of the model specification, allowing the reward to be objective or subjective depending upon the formulation. The probability of taking a specific action *a* in a particular problem state *s* is known as the policy, *p*(*a—s*), as this defines the action choice throughout the performance sequence. An MDP is specified over a state space *S*, which includes all possible configurations of the problem space, and an action set A, which includes all available actions (Figure 3). If the state space is not fully observable to the decision maker, the model is defined over a distribution of possible states and is referred to as a POMDP.

Central to the MDP is the definition of the Q function, which is the expected sum of discounted rewards obtained by taking action *a* while in state *s*:


(1) Q(s,a)=∑s∈′Sp(s|′s,a)(r(s,a,s)′+γ∑a∈′Ap(a|′s)′Q(s,′a)′)

where p(s|′s,a) is the transition function, specifying the probability of transitioning to a state *s′* given that action was taken in state *s*. Within the large brackets, *r*(*s*,*a*,*s*′) specifies, specifies the immediate reward for taking action *a* in state *s* and entering state *s′* while the second term represents the expected value of state *s′* over all possible actions, discounted by γ ∈ [0,1] to account for decreased value of future vs. immediate rewards.

When implemented to drive decision making for artificial agents, an optimal policy is frequently used in which at each decision point the action with the largest expected reward is taken. When used as a cognitive model, however, optimal decision making is not assumed and a “noise” parameter β is introduced to quantify the extent to which decisions are made optimally (Baker et al., 2009).


(2) $$\begin{array}{c} p\left(a|s\right)\infty exp\left(\beta Q\left(s,a\right)\right),\\ \beta \in \left[0,\infty \right) \end{array}$$

As β goes to infinity, this policy converges to the optimal policy, while as β goes to zero, the policy devolves into selection of actions from A uniformly at random.

The MDP measurement framework (LaMar, 2018) supports the development of cognitive profiles which might explain human behavior within a complex task. POMDPs can be interpreted as cognitive models in which behavior is driven by the actor’s goals and guided by their beliefs (Baker et al., 2011). As a cognitive model, the POMDP represents goals and motivation in the reward system and beliefs about the system dynamics in the transition functions. When the state space is not fully observed, the agent’s beliefs about the unobservable factors can be included as state variables within the state space itself. These beliefs and how they are updated can be used to model different perspectives and how learning occurs during interaction with the task. These properties make MDPs well-suited to differentiation of mindset behavior which is theorized to strongly impact goals, motivations, and beliefs. In addition to use as performance classifiers, the generative models allow us to test our theories about what mechanisms might be responsible for behavioral differences found in different mindsets through simulation of player performances. In this study we carefully examine performances simulated by fixed and malleable mindset models to better understand how our theories of action within the models translate to a small game task.

### The Cognitive Model

We used tenets of Mindset theory (see Dweck, 2006) along with evidence from a cognitive task analysis of the social simulation game run with young adults to operationalize behavioral differences based on having fixed or malleable cultural mindsets. Using a theory of mind based on an MDP framework (Baker et al., 2009) we built a cognitive model that included the three mechanisms: goals, initial beliefs and belief updating. In the MDP generating model, fixed players were modeled as focusing more on performance in terms of attaining the task goal of getting water and were only mildly curious about learning the cultural norms in the game (deemed as the confirmatory reasoning strategy). In essence, the fixed player begins the game with preset beliefs about the norms in this culture based on age. When the fixed player finds that NPC feedback does not match their beliefs they move on quickly and spend little time thinking about why the NPC responded this way – there is a tradeoff of efficiency and learning. On the other hand, the malleable mindset players were modeled with the focus being on learning the cultural norms of the game in addition to completing the task goal of finding water (as detailed earlier we deemed this the explore and learn strategy). The malleable player starts with more of an open mind and less strong preset beliefs about the norms of this culture and when faced with negative NPC feedback, spends more time investigating why this might be the case.

#### Goals

As part of a subjective cognitive model, the goals represented in the model are not purely extrinsic rewards, but include whatever factors drive decision-making for each type of person. Because one of the main differences between our fixed and malleable mindset models involves goals, it is theorized that fixed-mindset players will focus more on concrete task goals (i.e., finding water), while malleable-mindset players will be more motivated to understand cultural norms of the game. Thus, malleable-mindset players are modeled as having the primary goal of figuring out which age groups like to be treated formally in addition to the goal of finding water, whereas, the fixed-mindset players are modeled as primarily focusing on the goal of finding water, with mild curiosity about the game culture.

#### Initial Beliefs

It is theorized that fixed-mindset players will bring preconceived notions about cultural norms into a new culture. These could be based on their own native culture or stereotypes they have developed about non-native cultures. In either case, the player is likely to hold strong expectations about what is appropriate and how others will act and react. For malleable-mindset players it is theorized that they will enter a new culture with a relatively open mind about how people from this culture will behave and what practices are appropriate.

#### Belief Updating

In addition to differences in the initial beliefs brought into the scenario, it is theorized that fixed-mindset players will be resistant to changing their beliefs while malleable-mindset players will be more receptive to feedback that indicates their beliefs are incorrect.

### MDP Models for Cultural Mindset

Mathematically, each cognitive model is implemented in the state space, transition functions, and reward values of an MDP (details below). The exact configuration and parameter values of the models were determined based on an iterative simulation process in which a candidate model would be used to simulate data. Expert opinion was elicited to determine how well the simulated play records represented behavior that might be seen from fixed-mindset or malleable-mindset players, respectively, and the model was updated to better match the expert judgment, producing a new candidate model.

#### State Space as Beliefs

The MDP state space tracks the world as the player understands it. This includes: the status of the scenario, such as which NPC the player is interacting with, whether or not the player has received information about the location of the water, and the status of the player’s culturally specific beliefs (e.g., which NPCs like to be treated formally). All state-space variables are discrete integer values and are listed in Table 2.

**TABLE 2:** State space variables, their type and range, and the initial values for fixed and malleable mindset models.

State Variable	Type	Min	Max	Fixed Initial	Malleable Initial
OLD_LIKES_FORMAL	Cultural Norm Belief	−2	2	2	0
MlD_LlKES_FORMAL	Cultural Norm Belief	−2	2	1	0
YOUNG_LlKES_FORMAL	Cultural Norm Belief	−2	2	−2	0
IN_CONVERSATION	Situation Status	0	1	0	0
NPC AGE	Situation Status	0	3	0	0
GREETED	Situation Status	0	1	0	0
ASKED	Situation Status	0	1	0	0
NPC_HAPPY	Situation Status	0	1	0	0
GOT_WATER	Quest Status	0	1	0	0

The three Cultural Norm Belief variables represent the player’s mental model of which age classes like to be treated formally. They range from −2 (indicating that the player is certain the age group does not like to be treated formally), to 2 (indicating that the player is certain the age group does like to be treated formally). A value of zero means the player is not sure how the age group likes to be treated. The initial values, shown for the two models in the right-most columns, reflect that the fixed-mindset players start with a belief that old people like formality and young people dislike it. They are less certain about middle-aged people, but suspect that they like formality too. The malleable-mindset players, on the other hand, start out agnostic – signifying awareness they have not learned this culture yet.

Note that even the game-status variables are beliefs, in that they indicate the player’s internal representations. While the player is unlikely to be wrong about some, the model allows for any variable to be out of sync with the real world (e.g., whether or not the NPC is happy). Thus, there exists a parallel model that represents the actual world, known as the real-world model. This model contains the same state variables as the cognitive model, but they are set to objective reality.

#### Transition Function as Beliefs

In addition to the state space variables, the cognitive model encodes how those variables are expected to change when different actions are taken. For example, when a player greets an elder formally, they will expect the elder will become pleased (i.e., NPC_HAPPY → 1) *if and only if* they also believe that old people like formality (OLD_LIKES_FORMAL > 0). These changes to the world are encoded in transitions functions that describe how any of the available actions probabilistically affect the state variables.

We assume that both the fixed-mindset and malleable-mindset players share the basic reasoning that using the appropriate formality with an NPC will make them happy, and that a happy NPC is more likely to tell you where the water is. What differs in the transition functions between the fixed and malleable mindsets is how they expect their own beliefs to change. We model the fixed-mindset players as not expecting to learn from their NPC interactions unless they are completely uncertain of the correct formality. Thus, their belief is expected to change only if X_LIKES_FORMAL is set to zero. By contrast, the malleable-mindset players expect to learn unless their beliefs are maxed in the correct direction. Therefore, the malleable-mindset players might choose actions to increase their understanding of the culture, but the fixed-mindset players will not do so unless they are confused (X_LIKES_FORMAL = 0).

The player-model transition functions are, again, representing the player’s internal model or prediction of what might happen. The real-world models generate the actual outcome. This is where we encode the real cultural norms which specify what actions will make an NPC happy and when they will comply with a request for the location of the water. Further, the real-world model specifies how each type of player will actually update their beliefs. The fixed-mindset player is expected to rationalize unhappy NPCs rather than reason that their own beliefs about cultural norms might be wrong. We set up the model so that the stronger the belief held, the less likely the player will update it. Probabilities of updating the YOUNG_LIKES_FORMAL variable are shown in Table 3. For the fixed-mindset players, the probability of updating the belief increases when the feedback given is closer to their already held belief, as an implementation of confirmation bias. For malleable-mindset players, belief updating is most likely when they are uncertain, but is symmetric around the uncertainty peak. Thus, feedback that a young person likes to be treated formally would be equally likely to increase the YOUNG_LIKES_FORMAL variable whether the current belief is moderately positive or moderately negative. Note that for both models beliefs are maximally changed by 1 for each piece of feedback received. This models the idea that belief change is based on accumulation of evidence from the environment. The quantity of evidence needed for belief change could be modified by changing the range of the belief variables (e.g., ranging from −3 to 3 would require six pieces of feedback before any player changes from a completely incorrect belief to a strongly held correct belief). Table 3 does not include probabilities for changing belief at the current value of 2 because it is impossible to correctly update a belief that is already at the max correct value (2 for YOUNG_LIKES_FORMAL).

**TABLE 3:** Real-world model for belief updating of fixed-mindset and malleable-mindset players.

YOUNG_LIKES_FORMAL Current Value	Probability of Updating after Feedback	
	fixed-mindset	malleable-mindset
−2	0.20	0.30
−1	0.40	0.60
0	0.60	0.95
1	0.80	0.60

#### Goals

The MDP model goals are encoded by relative values of reward produced when particular conditions are met. “Rewards” can also be negative, which indicate costs. Both models include four reward conditions and a time cost. The function of the time cost is to prompt efficiency of action and prevent endless wandering through the problem space. The time cost is constant and occurs after each time slice. The quantity of the cost can represent motivation, as a larger cost will cause players to end quickly, perhaps even without achieving the positive rewards, while a smaller cost will encourage more exploratory behavior and greater persistence in achieving the maximal positive rewards. The rewards for the two models are shown in Table 4.

**TABLE 4:** Reward values for different achieved conditions for the two mindset models.

*Reward Label*	*Condition*	*Fixed*	*Malleable*
*Got the Water*	*GotWater* = = 1	5	*5*
*Bit of Beliefs*	3>∑_x∈{Y,M,O}_|LF(x)_F_|>0	0.5	1
*Moderate Beliefs*	6>∑_x∈{Y,M,O}_|LF(x)_F_|≥3	1.5	3
*Strong Beliefs*	∑_x∈{Y,M,O}_|LF(x)_F_|==6	3.5	6
*Time Cost*	Every move	−0.75	−0.5

*LF(x) is shorthand for X_LIKES_FORMAL while Y, M, O indicate Young, Middle, and Old.*

Except for the time cost, all rewards are given at the end of play. Thus, whatever conditions are achieved at the end will be rewarded. Both models include a reward of 5 for getting the water information. This fixed value gives a common scale for the relative worth of the remaining rewards. The belief-based rewards (rows 3–5) represent a discomfort with uncertainty and a value of “knowing” the cultural beliefs. These rewards are not based on knowing the correct answer, but instead on believing you know the correct answer. Stronger belief is quantified by the absolute value of the X_LIKES_FORMAL variables, where *LF(x)*_F_ is the value of the “LIKES_FORMAL” variable for the age-class *x* at end of the game and Y, M, and O stand for Young, Mid, and Old, respectively. The fixed-mindset model, which starts with moderate-to-strongly held beliefs, would already achieve a “belief reward” of 1.5 and be motivated primarily to get water and get out. Even if their certainty of the culture is lowered as they interact with the NPCs, the fixed-mindset people are more highly motivated by finding the water than by correcting those beliefs, which would give a max of 3.5 extra reward. The malleable-mindset model rewards strong belief even more highly, with full understanding of the culture worth more to the model than finding water. Also, the malleable-mindset model starts with X_LIKES_FORMAL values all set to zero, requiring the player represented by this model to take many actions to discover and confirm the correct cultural norms. In addition, the fixed-mindset model includes a greater cost for time, modeling less motivation overall to engage with the task.

### Simulation Study and Analyses

A simulation study was conducted to gather preliminary validity evidence for the modeling approach along with formative feedback for refining both tasks and models. Specifically, the simulation study was intended to examine (1) the extent to which the models generate behavior that we associate with each cognitive profile and (2) the recoverability of the profile classes using maximum likelihood estimation techniques.

#### Data Generation

The data were simulated to allow a direct comparison between fixed and malleable mindset play records. Most of the parameters of the MPD model are contained in the reward functions and transition functions. These parameters are set to create the two profiles (fixed and malleable) as described above. The single remaining parameter in the model allows for differing strategic ability (see LaMar, 2018) in a continuous β. Because we wanted to allow for differing strategic abilities, we drew 500 β values from a log-normal distribution, β∼lnN(0,1). Each β value was then used to simulate one fixed and one malleable mindset player, resulting in a pair of comparable simulated players of different mindsets. For each simulated player, the appropriate MDP model was used to simulate 100 independent play records (one full run of the game). Each of these records was simulated by starting in the game state defined by the model’s initial values (Table 2) and drawing an action from the action set based on the model’s defined policy (LaMar, 2018) and the player’s beliefs. The game state would then transition to a state drawn from each model’s real-world specification, and the process would repeat until the player drew a “STOP” action, which would end play. Because the MDP model is probabilistic, most simulated game plays were different, even when they started with the same parameter values.

## Analytic Plan

### Testing Behavioral Theory

To determine if the simulated play records correspond to the intended mindsets, we test: four criteria based on existing theory, the recoverability of the cultural mindsets based on simulation, and an exploratory hypothesis about choosing different NPCs. For each criteria we summarize below the theory on which the criteria is based. Unless otherwise noted, all count and score comparisons between records were first aggregated across the 100 simulation runs for each simulated player. The mean values for each of the 1000 simulated players (500 generated each for the malleable and the fixed mindset models, respectively) are then compared using the Welch two-sided *t*-test to allow for unequal variances. All analyses were completed using R.

### Criteria 1: Cultural Learning

We expected malleable mindset players would be more successful at learning cultural norms of the game. To test this criteria, learning is operationalized as the amount that the modeled belief variables (X_LIKES_FORMAL) move closer to the correct beliefs about the simulated culture during a complete game play. Each of the formality belief variables have a correct value (2 for Old and Young and −2 for Mid) and all belief changes within our model move these beliefs closer to the correct values. Thus, the learning score was the sum of the absolute value of the difference between the model’s initial and final beliefs over all three belief variables:


$$\sum_{X\in \left(Young,Mid,Old\right)}\left|LF{\left(X\right)}_{F}-LF{\left(X\right)}_{0}\right|,$$

where LF(X)_t_ is the value of the “LIKES_FORMAL” variable for the age-class X at time t. *F* is the time point at which the player ended the game and 0 is the time point at the beginning of the game. We then compared mean learning scores for fixed and malleable mindset players. Note that because the two models start at different initial beliefs (Table 2), they have different amounts of potential learning. The fixed mindset models have a max learning of 7 (0 + 3 + 4) while the malleable mindset models have a max learning of 6 (2 + 2 + 2).

### Criteria 2: Efficiency and Time to Task Completion

We expected fixed mindset players to have fewer conversations and fewer turns per conversation than malleable players. We compared the fixed and malleable models on the mean number of conversations they engaged in for each game and the mean number of actions per conversation across the full 100 runs of gameplay.

### Criteria 3: Persistence

We expected fixed mindset players to be less resilient to failure and therefore more likely to give up in the process of achieving the task goal of finding water and more likely to switch conversational partners when not immediately successful. Game-level quitting is operationalized as exiting the game without finding water after fewer than 3 conversations. Conversational quitting is operationalized as leaving a conversation without gaining information about the water after fewer than 4 dialog turns. For both behaviors we test differences between models using the χ_2_-test over total conversations or total games generated by the two models.

### Criteria 4: Responsiveness to Feedback

We expected fixed mindset players to be less likely to change their behavior after receiving negative feedback. We operationalize responsiveness at the conversation level as use of incorrect formality followed by at least 2 uses of the correct formality. Because one cannot be responsive to negative feedback if none is received, we calculate responsiveness as the proportion of conversations in which the player showed responsiveness out of conversations in which at least one incorrect formality was chosen. Behavioral differences were tested using the χ_2_-test over total conversations generated by the two models.

### Cultural Mindset Classification

Testing the criteria listed above allowed us to evaluate whether the models are generating data in accordance with our intended theory. As a proposed model for classifying human behavior, however, it is essential to evaluate the ability of the MDP approach to correctly classify play records by cultural mindset. Thus, in the next part of this study we evaluate model recovery by gathering all simulated play records into a single data set and classifying them as either fixed or malleable using maximum likelihood estimation. The log likelihood of each play record is calculated for both models by summing the log of the model’s predicted probability of taking each action in the play record, given the current state over all actions in the record. Because the β value essentially adds in performance error (with lower values of β introducing more error), the generating β value can have a large effect on the resulting records. Thus, we also evaluate classification accuracy as a function of β.

### Exploratory Analysis

Because the age of the NPCs was visually apparent during real-world gameplay and players may have pre-existing normative beliefs about age, we explored whether the models would simulate differences in preferences for interacting with NPCs based on age.

## Results

### Criteria 1: Cultural Learning

**FIGURE 4:**
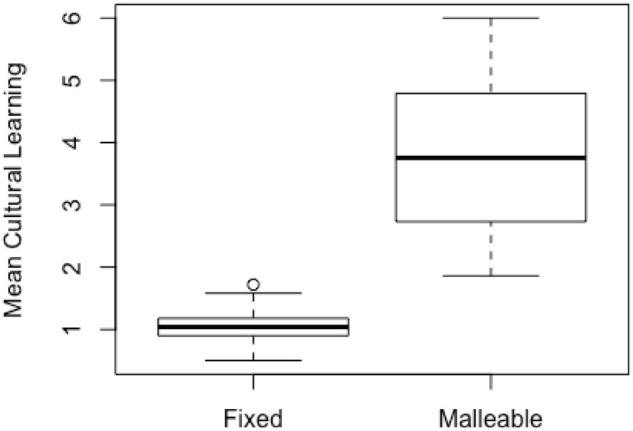
Mean amount of cultural norm learning, as quantified by the difference between correctness of the initial and final formality beliefs, clustered by generating model. Each box represents the interquartile range with a median line and the whiskers represent 95% range given normality.

Records generated by the malleable mindset models showed significantly higher learning of cultural norms as seen in Figure 4. The mean learning across all games for the malleable mindset was 3.8 (*SD* = 1.17) vs. a mean of 1.0 (*SD* = 0.20) learning for the fixed mindset models [*d* = 1.71, *t* (529.31) = 52.04, *p* < 0.001]. We note that although the fixed mindset had larger potential learning, the mean cultural learning for the fixed mindset players was significantly lower than the mean learning produced by the malleable mindset model.

### Criteria 2: Efficiency and Time to Task Completion

**FIGURE 5:**
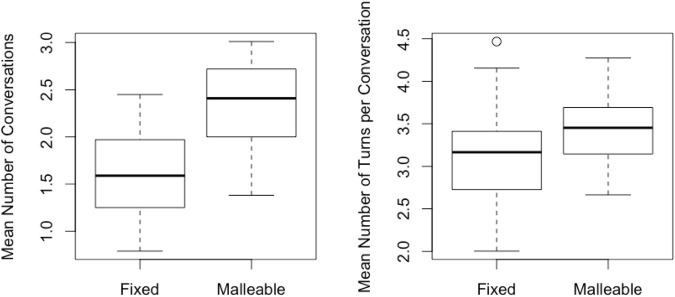
Differences in gameplay based on generating model. On the left the mean number of conversations per game played; on the right the mean number of turns taken in each conversation. Each box represents the interquartile range with a median line and the whiskers represent 95% range given normality.

As shown in Figure 5, the mean number of actions per conversation generated by the malleable model (*M* = 3.43, *SD* = 0.35) were significantly higher than the fixed model (*M* = 3.07, *SD* = 0.49) [*d = 0.78, t* (910.85) = 13.4, *p* < 0.0001]. Moreover, additional evidence to support this criteria was found in the mean difference in number of conversations started: *d* = 1.37, *t*(994.78) = 29.7, *p* < 0.0001, indicating that the malleable model generated significantly more conversations (*M* = 2.4, *SD* = 0.42) compared to the fixed model (*M* = 1.61, *SD* = 0.39).

### Criteria 3: Persistence

We assessed persistence by how much the fixed and malleable players ended conversations before achieving the task goal during gameplay. Game records from the fixed mindset model displayed conversational quitting in 42.9% of conversations, while the records from the malleable mindset only displayed conversational quitting in 25.3% of conversations. These proportions were found to be highly significantly different using a *χ*^2^ test of equality [*χ*^2^ (1) = 6745.6, *p* < 0.001]. Game quitting behavior followed a similar pattern, with records generated by the fixed mindset model ending the game early 30.0% of the time while the malleable model quit the game 15.0% of the time [*χ*^2^ (1) = 3206.1, *p* < 0.001].

### Criteria 4: Responsiveness to Feedback

The malleable mindset simulations showed responsiveness to feedback in 24.1% of their conversations as compared to 16.2% of the fixed mindset conversations. This difference was highly significant [*χ*^2^ (1) = 1786.7, *p* < 0.001]. Because it was not possible to be responsive to negative feedback if no negative feedback occurred, the analysis was rerun on the subset of conversations in which at least one incorrect formality was used. For this subset, the malleable model showed responsiveness in 31.3% of conversations while the fixed model showed responsiveness in 22.3% of conversations [*χ*^2^ (1) = 1427.2, *p* < 0.001]. Note that malleable mindset conversations were slightly more likely to receive negative feedback, with 77% of malleable conversations receiving negative feedback compared to 73% of conversations from the fixed mindset model receiving negative feedback.

### Cultural Mindset Classification Analysis

The MDP cultural mindset classification correctly classified the generating model for 83% of the simulated game records. The confusion matrix (Table 5) shows that records generated by the fixed mindset model were more likely to be correctly classified than records generated by the malleable mindset model (88% and 78%, respectively).

**TABLE 5:** Confusion matrix for the MDP mindset classifier.

	Classified as	
Generating model	Fixed	Malleable
Fixed	0.88	0.12
Malleable	0.22	0.78

**FIGURE 6:**
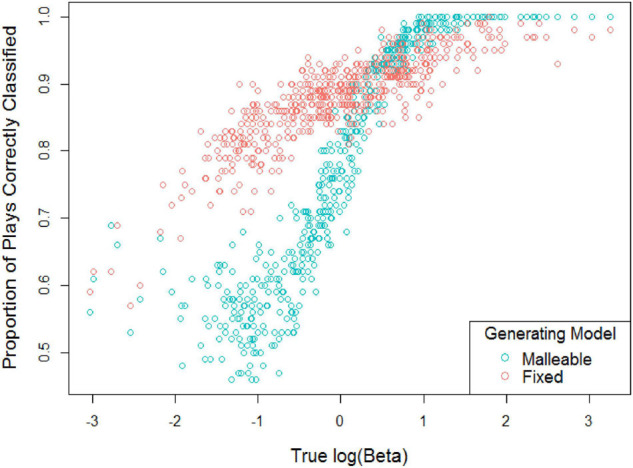
Cultural mindset classification accuracy across replicated runs by simulated player.

In general, the records were more likely to be classified as Fixed, with 55% of all records classified as fixed and a false positive rate for Fixed classification of 22%. To understand the possible interaction between β-values and classification accuracy, we aggregated correct classification by simulated player and plotted the proportion of correctly classified game records as a function of the simulated player’s log(β) value, shown in Figure 6.

Interestingly the pattern of classification accuracy by log(β) is starkly different between the two different mindset models. For the malleable mindset, players with low log(β) (indicating that they were making less-than-optimal decisions according to the malleable mindset model), were very difficult to classify, with classification accuracy hovering just above the chance 0.5 mark up to log(β) = −0.5. As log(β) increases, however, the malleable mindset records become very recognizable, with accuracy rising to 1.0 by log(β) = 1.5. In contrast, records generated with the fixed mindset model are classifiable even at low log(β), but never rise to completely accurate. This unexpected finding may be an artifact of the simulation itself, or it may indicate an important difference in how recognizable behaviors from the different mindsets are, given other individual differences.

### Exploratory Analysis of Choosing NPCs

**FIGURE 7:**
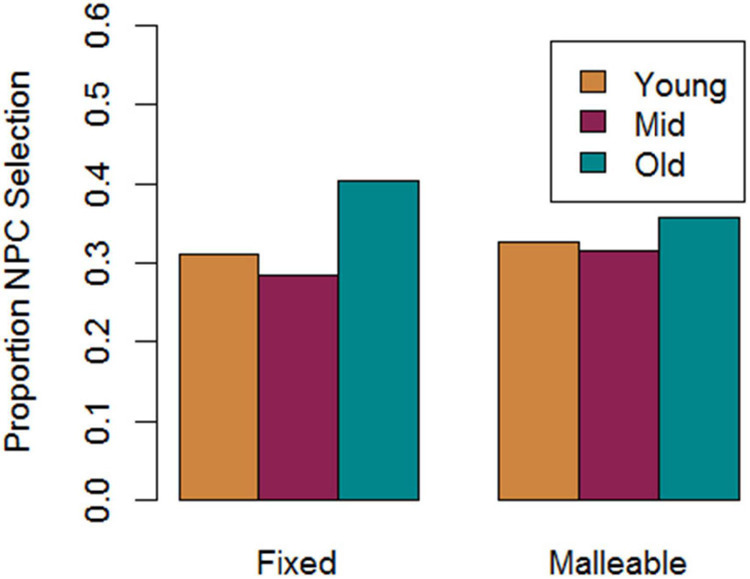
Proportion of conversations for which different age category NPCs were selected.

We explored how the fixed and malleable models interacted with different characters during gameplay. Because real-world players may have pre-existing normative beliefs about age we explored whether the models would simulate differences in preferences for interacting with NPCs based on age. As shown in Figure 7, the proportions of NPCs chosen were significantly different (*χ*^2^ = 440.8, df = 2, *p* < 0.001) with the fixed mindset model showing a fairly strong bias toward selecting older NPCs, while the malleable mindset model generated records that were more evenly selected from the three available NPC age classes. Based on aiming to achieve the goal of getting water we’d expect fixed players to feel that older NPCs may be the safest choices to approach because players may expect that they will prefer formality when asking for water. Additionally, as older NPCs will reinforce their prior beliefs, the fixed mindset players may be more likely to return to them rather than risking conversations with other NPCs.

## Discussion

The goal of the present work was to use mindset literature to develop theoretical cognitive profiles for behavior during a social simulation game. We aimed to simulate how having more fixed or malleable mindsets impact learning and performance, specifically during a cross-cultural innovative assessment. We used an MDP approach to first simulate behavior based on fixed and malleable profiles. We then used the simulation results to: provide validity evidence based on testing behavioral criteria, investigate the MDP model classification for fixed and malleable players as well as exploring differences in fixed and malleable interactions with different non-player characters (NPCs). We found evidence to support the validity of our models.

First, our results indicate that compared to fixed players, malleable players did learn the culture faster as expected. This result is likely to be due to the greater value our malleable models put on learning. Note that the fixed models put a high value on task completion, which would be aided by learning the culture, yet this did not translate into equal learning. We also found that malleable players engaged in significantly more actions, conversations, and dialog during the game than did fixed players – with the largest difference in the mean number of dialog actions. This indicated that malleable players spent more time engaging with NPCs in conversations and in greeting and asking for water, supporting the criteria that malleable players should still value the success of getting the water but not at the expense of learning about the culture. Moreover, we found the percentage of quitting behavior among the fixed players to be significantly higher than the malleable players and subsequently, the percentage of times that fixed players quit the game early was also significantly higher than for malleable players. In terms of responding to negative feedback, we found evidence that fixed players may discount information that does not align with their theories in that relative to malleable players, they were significantly less responsive and less likely to change their behavior after receiving negative feedback from NPCs.

By exploring differences in the proportion of conversations players had with different NPCs, we also found evidence to support players’ preset beliefs about age and NPC greeting preferences. This was observed in differences in selection of older NPCs between fixed and malleable players. The idea was that fixed players may have the preset belief that older NPCs will prefer formal greetings, so they seek to collect support for this theory whereas, malleable players will be more motivated to explore and learn. Indeed, we observed a significantly greater preference for the older NPC among the fixed players. Additionally, there was more even selection from the three available NPC age classes in the malleable players, providing support for the theory that malleable players value exploration and learning more than fixed players in this context.

We found that our MDP classifier was acceptable as it correctly classified 83% of our simulated records and that, in general, records were more likely to be classified as fixed. In turn, we observed an interaction between the ability parameter and classification accuracy. At the low end of the ability parameter range, malleable players were difficult to classify but as ability increases classification accuracy of malleable players becomes very high. At present, it is unclear whether this finding is an artifact of the model development but these results were interesting and should be explored further in the future – if meaningful, this finding has implications for the interaction between a general ability parameter (e.g., intelligence, cognitive control, planning ability) and cultural mindset and how this may impact real world behavior and performance.

### Implications

Based on previous research, we know the broad impact of the benefits that cross-cultural competence brings in a variety of situations, including enabling ease of mutual understanding and trust and improving a variety of educational, health, and interpersonal outcomes (Berry et al., 2002; Stahl et al., 2009; Lim, 2014; Ashkinazy, 2017). Through theory, simulation, and behavioral criteria testing, our work demonstrates the impact that having different cultural mindsets might have on performance in terms of the ways people approach the process of navigating cross-cultural situations. Cultural mindset helps clarify the ways that differences in the processes by which people approach cross-cultural situations matter. As such, these findings could lead to interventions that improve cultural receptiveness and understanding in a range of sectors and fields.

In education, there has been a growing concern and recent discussion on topics of cultural awareness, cultural sensitivity, and diversity and inclusion (Bowman, 2010). Previous research finds that implicit bias among teachers has negative consequences for minority students in terms of educational outcomes and disciplinary actions (Gilliam et al., 2016; Riddle and Sinclair, 2019). Finding ways to promote better understanding and more empathic concern in teachers helps to reduce bias (Pettigrew and Tropp, 2008) and improves mutual trust and understanding among teachers and students (Okonofua et al., 2016). Additionally, cross-cultural competence is an important skill for effective and culturally responsive teaching in terms of boosting educational outcomes while decreasing inappropriate placement and referrals (see Gay, 2000; Goe et al., 2008; Lim, 2014). In fact, students who have teachers with whom they can identify through mutual trust and understanding are more likely to graduate high school and attend college (Gershenson et al., 2017, 2018). These findings underscore the importance of cultural sensitivity among teachers as well as the potential impact of cultural mindset in education. Efforts to improve cultural mindset, including increasing cultural awareness and perspective taking in order to understand and be inclusive among different cultural backgrounds can help make teachers more informed, relatable, and effective.

In healthcare, improving cultural mindset in terms of increasing cultural awareness, perspective taking, and the rapport building should help practitioners better serve clients. Previous research finds that forms of bias underlie many health disparities for minorities and lower SES individuals based on access to care, and gaps in quality of care (National Center for Chronic Disease Prevention and Health Promotion, 2016; also see Smedley et al., 2003). As such, finding ways to improve the process by which people navigate cross-culturally diverse interactions in healthcare is critical for helping to improve healthcare outcomes (see Ashkinazy, 2017). Cultural mindset is one approach that future research can use to uncover ways to produce a health care system with more culturally competent providers.

In business, cultural awareness, perspective taking, and building rapport are essential parts of 3C that could be the focus in order to improve cultural mindset within individuals and organizations. Gaps in productivity arise from barriers to shared understanding, and failures to exercise the range of skills and abilities that make diverse groups valuable (DiStefano, 2003). Highly- diverse culturally- competent teams can provide greater satisfaction and more creativity (Stahl et al., 2009), however, diverse teams perform best contingent upon acknowledging and working through differences and establishing team norms (DiStefano, 2003). Individuals and organizations in the business sector can use cultural mindset to improve cross-cultural competence and performance by strengthening mutual trust and understanding, fostering openness to feedback, and willingness to learn.

Another area where this work would be of great importance is in the military. Due to the fact that engagement and cross-cultural interaction is becoming the norm for soldiers who are deployed (see McCloskey et al., 2010), having a better understanding of the process and mechanisms involved in the architecture of cross-cultural competence is key. Cultural mindset provides a theoretical underpinning for investigating the processes that allow people to navigate cross-cultural interactions successfully or unsuccessfully. For these reasons cultural mindset is amenable and applicable for research and interventions tailored for developing and improving cross-cultural interaction at the tactical level.

### Limitations

It is important to acknowledge a few limitations regarding the use of mindset theory to frame our exploration of cultural mindset. First, in mindset theory (Dweck, 2006), mindset is characterized as a continuum of beliefs ranging from more fixed to more malleable and we acknowledge that we characterized these in a more categorical way in order to explore cognitive profiles of cultural mindset in the context of cross-cultural learning and performance. We characterized those with fixed cultural mindsets as having cultural understanding and attitudes that are unlikely to change – extended to thinking about cultural norms and culturally appropriate behavior rigidly. That is, those with fixed cultural mindsets were hypothesized to be less likely to update their prior beliefs based on new experiences. We acknowledge that there might be several ways to model this based on mindset theory. One way this could be modeled is that in spite of many new experiences fixed cultural mindset people could have a harder time using the information to actually update their prior beliefs. Or, perhaps the new information is discounted in a way that would not be the case for a malleable cultural mindset person. It is also possible that fixed cultural mindset people simply need to accumulate many more new experiences in order to update their prior beliefs as compared to a malleable cultural mindset person. We aimed to incorporate this level of depth into our models as much as possible but there remain many ways to cognitively model fixed and malleable cultural mindsets based on using mindset theory and those outlined here are by no means exhaustive. Future work should further explore these and other potential ways of characterizing cognitive profiles in order to gain a more nuanced understanding of mindset effects.

Second, there have been recent concerns and discussion in the field about the replicability of mindset’s effects on behavior and performance, typically in academic achievement domains (Holden et al., 2016; Sisk et al., 2018; Foliano et al., 2019; Martinez et al., 2020; Holden and Goldstein, 2021 manuscript; also see Yeager et al., 2019) and its relative importance in terms of whether the construct is overstated for its role in motivation and performance enhancement (Moreau et al., 2019; Burgoyne et al., 2020). These concerns are critical for thinking about the generalizability of effects based on mindset theory. We used mindset theory as a framework for developing the cognitive profiles in our models to be applied in the novel domains of cross-cultural learning and performance. However, it is critical to acknowledge the current concerns in the field and how this might be important for thinking about work employing mindset theory. For these reasons, we urge the continued investigation of boundary conditions of mindset theory and mindset effects in future work – considering both its role in and importance for predicting behavior in a variety of performance domains.

## Conclusion

Cultural mindset provides a new framework for research in improving our understanding and development of cross-cultural competence for scientists, educators, business people, healthcare professionals, military personnel, and beyond. Taken together, our simulation study and results reveal evidence to support fixed and malleable cultural mindsets as an important theoretical framework for investigating cross-cultural learning and performance. Likewise, utilizing the MDP approach also proved fruitful for generating a nuanced and rich analog for decision-making during a cross-cultural innovative assessment. As such, future directions should explore the concept of cultural mindsets in greater detail. Additionally, the potential interaction we observed between the ability parameter and cultural mindset should be further investigated, and how this may contribute to the understanding of cross-cultural performance in terms of action, learning, and decision-making.

## Data Availability Statement

The software, simulated data, and the full model specifications will be made available in supplemental online material pending approval from the Educational Testing Service. Requests to access these materials should be directed to LH, lholden@memphis.edu.

## Author Contributions

LH, ML, and MB developed the cognitive profiles from cognitive labs. LH wrote the manuscript and advised development of the Markov models. LH and ML developed research questions and each ran portions of the analyses. ML developed the Markov models and assisted in writing portions of the manuscript. MB assisted in development of the Markov models and revising portions of the manuscript. All authors contributed to the article and approved the submitted version.

## Conflict of Interest

The authors declare that the research was conducted in the absence of any commercial or financial relationships that could be construed as a potential conflict of interest.

## Publisher’s Note

All claims expressed in this article are solely those of the authors and do not necessarily represent those of their affiliated organizations, or those of the publisher, the editors and the reviewers. Any product that may be evaluated in this article, or claim that may be made by its manufacturer, is not guaranteed or endorsed by the publisher.

## Footnotes

1 We note here that Adair et al. (2013) were the first to our knowledge to use the term “cultural mindset” however, their use of the term was different. Specifically, Adair et al. argue that in order for a team to develop shared values, individuals must adjust their motivation and work to initiate shared values. Suggesting, shared values become integrated into each individual’s *malleable identity* that is *activated* in team settings. Adair et al. also mention what is called cultural mindset, stating “…individuals can adjust their motivational values in different situations… [and] literature on bicultural identity shows that individuals can shift their cultural mind-set depending on the situation…” Here, Adair et al. suggest that how people think, feel and behave can be influenced by different cultural identities. In turn, individuals’ cultural values and motivations can be altered or adjusted depending on the situation at hand – deemed as the shifting of one’s “cultural mind-set.”
